# Genome-Wide Association Mapping Reveals the Genetic Control Underlying Branch Angle in Rapeseed (*Brassica napus* L.)

**DOI:** 10.3389/fpls.2017.01054

**Published:** 2017-06-19

**Authors:** Hongge Li, Liping Zhang, Jihong Hu, Fugui Zhang, Biyun Chen, Kun Xu, Guizhen Gao, Hao Li, Tianyao Zhang, Zaiyun Li, Xiaoming Wu

**Affiliations:** ^1^Oil Crops Research Institute of the Chinese Academy of Agricultural Sciences, Key Laboratory of Biology and Genetic Improvement of Oil Crops, Ministry of AgricultureWuhan, China; ^2^National Key Lab of Crop Genetic Improvement, National Center of Crop molecular Breeding, National Center of Oil Crop Improvement, College of Plant Science and Technology, Huazhong Agricultural UniversityWuhan, China

**Keywords:** *Brassica napus* L., branch angle, plant architecture, association mapping, candidate-genes

## Abstract

Plant architecture is vital not only for crop yield, but also for field management, such as mechanical harvesting. The branch angle is one of the key factors determining plant architecture. With the aim of revealing the genetic control underlying branch angle in rapeseed (*Brassica napus* L.), the positional variation of branch angles on individual plants was evaluated, and the branch angle increased with the elevation of branch position. Furthermore, three middle branches of individual plants were selected to measure the branch angle because they exhibited the most representative phenotypic values. An association panel with 472 diverse accessions was estimated for branch angle trait in six environments and genotyped with a 60K *Brassica* Infinium® SNP array. As a result of association mapping, 46 and 38 significantly-associated loci were detected using a mixed linear model (MLM) and a multi-locus random-SNP-effect mixed linear model (MRMLM), which explained up to 62.2 and 66.2% of the cumulative phenotypic variation, respectively. Numerous highly-promising candidate genes were identified by annotating against *Arabidopsis thaliana* homologous, including some first found in rapeseed, such as *TAC1, SGR1, SGR3*, and *SGR5*. These findings reveal the genetic control underlying branch angle and provide insight into genetic improvements that are possible in the plant architecture of rapeseed.

## Introduction

In nature, a particular plant specializes its architecture and corresponding function. For crops, such as rapeseed (*Brassica napus* L.), the desirable architecture is able to produce high grain yields (Wang and Li, 2008). Shoot branching, such as branch angle (BA), is a principal factor in plant architecture (Ariyaratne et al., 2009). Plant density is a vital environmental factor influencing the plant architecture (Diepenbrock, 2000), and results in the capacity to bend the branches to suitable angles for increasing the photosynthetic efficiency. Mechanical harvesting, which is affected by many aspects especially shoot branching, is an inevitable option for the rapeseed industry in the future because of the resulting decreases in required labor resources. A higher plant density with decreased branching angle would produce the highest mechanical seed yields in rapeseed (Kuai et al., 2015).

Essentially, branch angle is one of the ways to adapt to diverse environmental conditions through gravitropism in plants, whereas gravitropism is achieved through asymmetric distribution of the auxin concentration (Roychoudhry and Kepinski, 2015). Considerable genes modulating branch angle have been identified in plants. In rice, *lazy1* was insensitive to gravity stimuli and exhibited a prostrate morphology due to impaired polar auxin transport (Li et al., 2007), and then, the orthologs of *LAZY1* in *Arabidopsis* and maize were characterized and cloned (Dong et al., 2013; Yoshihara et al., 2013). In contrast to the *lazy1* mutant, the *tac1* mutant had an almost vertical tiller angle in rice (Yu et al., 2007), and *TAC1* played an antagonistic role to *LAZY1*, although they belong to the same *IGT* gene family (Dardick et al., 2013). Furthermore, the *TAC1* orthologs in maize and *Miscanthus* were reported to regulate leaf angles (Ku et al., 2011; Zhao et al., 2014). A series of *sgr* mutants have been identified and manifested as a defective in gravitropic response that lead to a discrepant branch growth angle in *Arabidopsis* (Fukaki et al., 1996; Yamauchi et al., 1997). For instance, the normal endodermis, where gravity-sensing cells are located, was absent in the hypocotyls and inflorescence stems of *sgr1* and *sgr7* mutants (Fukaki et al., 1998). Furthermore, aberrant vacuoles affect amyloplast accumulation in the tissues of *sgr2*–*5* mutants (Hashiguchi et al., 2013). The other auxin homeostasis genes were also reported as participating in branch angle regulation, for example, gravity-induced PIN3 polarization diverts the auxin flow to mediate the asymmetric distribution of auxin for shoot bending (Rakusova et al., 2011).

A genome-wide association study (GWAS) has emerged as a powerful approach for dissecting important causal loci that correlated with complex traits. An excellent opportunity for insight into the genetic basis of agronomic traits at the DNA level for rapeseed is provided by the development of a 60K *Brassica* single nucleotide polymorphism (SNP) Infinium array (Edwards et al., 2013) and the completion of *B*. *napus* genome sequencing (Chalhoub et al., 2014). Thus, association mapping has been widely implemented for numerous traits in rapeseed in recent years, such as the seed weight, plant height, oil content, and clubroot resistance (Cai et al., 2014; Li et al., 2014, 2016a,b). Nevertheless, an association analysis for branch angle in rapeseed has not been well elucidated. Liu et al. (2016) detected 25 significantly associated quantitative trait loci (QTLs) and identified three candidate genes, including *LAZY1*, for branch angle across 143 rapeseed accessions. As reported by Sun et al. (2016a), 56 loci significantly associated with branch angle among 520 rapeseed accessions were confirmed, and many candidate othologs were detected, such as *LAZY1, SGR2*, and *PIN3*. However, some vital potential genes for branch angle, such as *TAC1, SGR1, SGR3*, and *SGR5*, have not been detected and remain to be further mined.

In this study, a massive phenotypic identification of branch angle was conducted in the association mapping of a population of 472 diverse rapeseed accessions in six different environments; the association mapping population was genotyped with a high-through 60K SNP array. Genome-wide association analysis was performed using two models, mixed linear model (MLM) and multi-locus random-SNP-effect mixed linear model (MRMLM), and 46 and 38 loci significantly associated with branch angle were mined, respectively. Subsequently, considerable highly-promising candidate genes were identified by annotating against *Arabidopsis thaliana* homologous. These findings will sharpen our understanding of genetic mechanisms underlying branch angle and will provide insight into genetic improvements that are possible for the plant architecture of rapeseed.

## Materials and methods

### Plant materials and field experiments

A panel of 472 rapeseed accessions collected worldwide and stored at the National Mid-term Gene Bank for Oil Crops of China was used for association analysis in this study. The informations of inbred lines about their origin and germplasm type have listed in a previous report (Li et al., 2014).

Field experiments were implemented in six environments across three growing seasons. During the 2013/2014 growing season, the association population was grown at Yangluo (114.50°E, 30.38°N) in Hubei province, which is referred to as E1; during the 2014/2015 growing season, the experiment was conducted at Wuhan (113.68°E, 30.58°N) and Yangluo, which are both in Hubei province, and are referred to as E2 and E3, respectively; and during the 2015/2016 growing season, the association panel was cultivated at Wuhan, Yangluo, and Changsha (113.00°E, 28.22°N, in Hunan province), and are referred to as E4, E5, and E6, respectively. A randomized complete block design with three replicates was adopted in each environment. Each plot contained two rows and 12–15 plants in each row, the distance between plants was 0.2 m within each row, and the space between rows was 0.3 m.

### Trait measurements and statistical analysis

Forty randomly-selected accessions were considered as a sub-panel for observing the positional variation trend of branch angle and for determining the appropriate measurement region on individual plants. In the field, five typical plants in each plot were selected to identify the branch angle at 6 weeks after pollination. The branch angle was defined as the angle between the main stem and its branch and measured by a digital protractor. In each plot of the sub-panel, the branch angle value was obtained by measuring all of the branches of five individual plants. In association panel, the values of the middle three branches of a plant were recorded as the individual plant branch angle value, and the average value of five plants in a plot represents the phenotypic data of a line in this plot.

The broad-sense heritability was estimated according to the following equation: *H*^2^ = δ_*g*_^2^/(δ_*g*_^2^ + δ_*ge*_^2^/*n* + δ_*e*_^2^/*nr*), where δ_*g*_^2^, δ_*ge*_^2^, δ_*e*_^2^, *n*, and *r* represent the genetic variance, the interaction variance between genotypes and environments, the error variance, the number of years/locations, and the number of replicates within each environment, respectively. For the branch angle trait, the variance components and best linear unbiased predictors (BLUP) of the multi-environment for each line were estimated using the lme4 package in R software based on a linear model (Merk et al., 2012). The final trait values for association analysis included the BLUP-value and single environment phenotypic data of each accession. The frequency distribution, correlation analysis, and comparative analysis were performed using R software.

### Genotype data acquisition

In previous reports, detailed descriptions about the process of SNP genotyping and mapping are provided, as are analyses of population structure and linkage disequilibrium (LD) (Li et al., 2014; Wang et al., 2016a).

In brief, the raw SNP data generated from the *Brassica* 60K Illumina® Infinium SNP array were clustered and automatically called using Illumina BeadStudio genotyping software. Subsequently, 26,841 high-quality SNPs with minor allele frequency (MAF) of more than 0.05 were retained for further analysis. In order to mapping the SNP to an exact position of the reference genome, a BLAST search (Altschul et al., 1990) was performed against *B. napus* genome sequences (Chalhoub et al., 2014) using the SNP sequences. Only the top and unique blast-hits were reserved.

Eventually, 19,945 SNPs were selected for principal component analysis (PCA), and a relative kinship and population structure analysis. The GCTA tool was used to construct a P matrix of PCA (Yang et al., 2011), SPAGedi software was served to build a K matrix of relative kinship (Hardy and Vekemans, 2002), STRUCTURE v2.3.4 was employed to infer a Q matrix of population structure (Pritchard et al., 2000) and TASSEL 5.0 was used to calculate LD (Bradbury et al., 2007).

### Haplotype block structure analysis

The haplotype block structure across 472 rapeseed accessions with the ultimately selected 19,945 SNPs was evaluated using Haploview v4.2 software (Barrett et al., 2005). The analysis referred to the definition of “strong LD” by Gabriel et al. (2002), i.e., the upper minimum of the confidence interval was 0.98 and the lower was 0.7. Furthermore, the fraction of strong LD in informative comparisons must be at least 0.95. Since Haploview software would ignore pairwise comparisons if the distance between markers was beyond 500 kb following the default parameter, to estimate all marker pairs, especially for a strong LD between markers above 500 kb, this setting was adjusted to zero.

### Genome-wide association study

The GWAS was implemented using two methods: a MLM (Yu et al., 2006) and a MRMLM (Wang et al., 2016b). The Q+K model, one of the MLMs, including both a fixed effect as the population structure matrix (Q) and a random effect as the kinship matrix (K) was adopted as the optimal model and was performed using TASSEL 5.0 software (Bradbury et al., 2007). An MLM can be described by the following matrix notation: *y* = *X*β + *Zu* + *e*, in which *y* is the phenotype; *X* is the genotype; β is a vector containing the fixed effects, including the genetic marker and the population structure (Q); *Z* is the relative kinship matrix; *u* is a vector of random additive genetic effects; and *e* is the unobserved vector of the random residual. The threshold of significant association between a trait and the SNPs in the MLM was *p* < 1.0 × 10^−3^ [i.e., −log_10_(*p*) = 3.0], which has been broadly adopted in the literature (Cai et al., 2014; Hatzig et al., 2015; Raman et al., 2015). The GWAS results were visualized with Manhattan and quantile-quantile (Q-Q) plots that were yielded from the qqman package in R software (Turner, 2014).

An MRMLM, which would improve the power and accuracy of the GWAS, was employed using the R package mrMLM, and the critical log of odds (LOD) score was set as 2.5 (Wang et al., 2016b).

The total phenotypic variation that was explained by the significant SNPs in the best fitting multiple regression model was estimated using the “stepAIC” function from the MASS package in R (Ihaka and Gentleman, 1996).

### Candidate genes identification

Two methods were performed to ascertain the region where the potential candidate gene was situated. The first method was based on a definition of the QTL: a locus showing true marker-trait association should harbor at least two SNPs with *p*-values above the threshold in a 1.5 Mb region (Wang et al., 2016a). Then, for the SNPs that could not be assigned to any QTL, the LD blocks where the associated SNPs were located, in which flanking markers had strong LD (*r*^2^ > 0.4), were regarded as the candidate gene regions. If the SNPs were also not located in the LD blocks, the 100 kb region centered on an unassigned SNP was considered as the potential candidate gene interval. An LD block analysis was performed using Haploview v4.2 with the default settings (Barrett et al., 2005).

To predict the function of candidate genes, a functional annotation was implemented. First, the protein sequences coded by candidate genes within the definitive region were extracted by referring to the annotation information for the *B. napus* “Darmor-*Bzh*” genome (http://www.genoscope.cns.fr/brassicanapus, Chalhoub et al., 2014). Later, the BlastP program was run against *Arabidopsis* protein sequences with the E-value ≤ 1E-10; then, the candidate genes were functionally annotated using the top hit of *Arabidopsis* homologous genes. Based on the research progress of branch angles, the orthologous genes involved in gravitropism and auxin transport were focused on.

### Quantitative real-time PCR analysis

To validate the expression level of candidate genes between extremely large and small branch angle accessions, four identified candidate genes were randomly selected to perform the quantitative real-time PCR (qRT-PCR) analysis. Gene-specific primers were designed using Primer-BLAST (https://www.ncbi.nlm.nih.gov/tools/primer-blast). For each accession, three middle branches at three weeks after pollination were harvested to extract total RNA. The procedure of total RNA extraction, cDNA synthesis, qRT-PCR amplification, and candidate genes expression analysis were as previously described (Yan et al., 2016). Each sample was examined in three independent biological replicates with three technical replicates.

## Results

### Positional variation of the branch angle on individual plants

**Figure 1:**
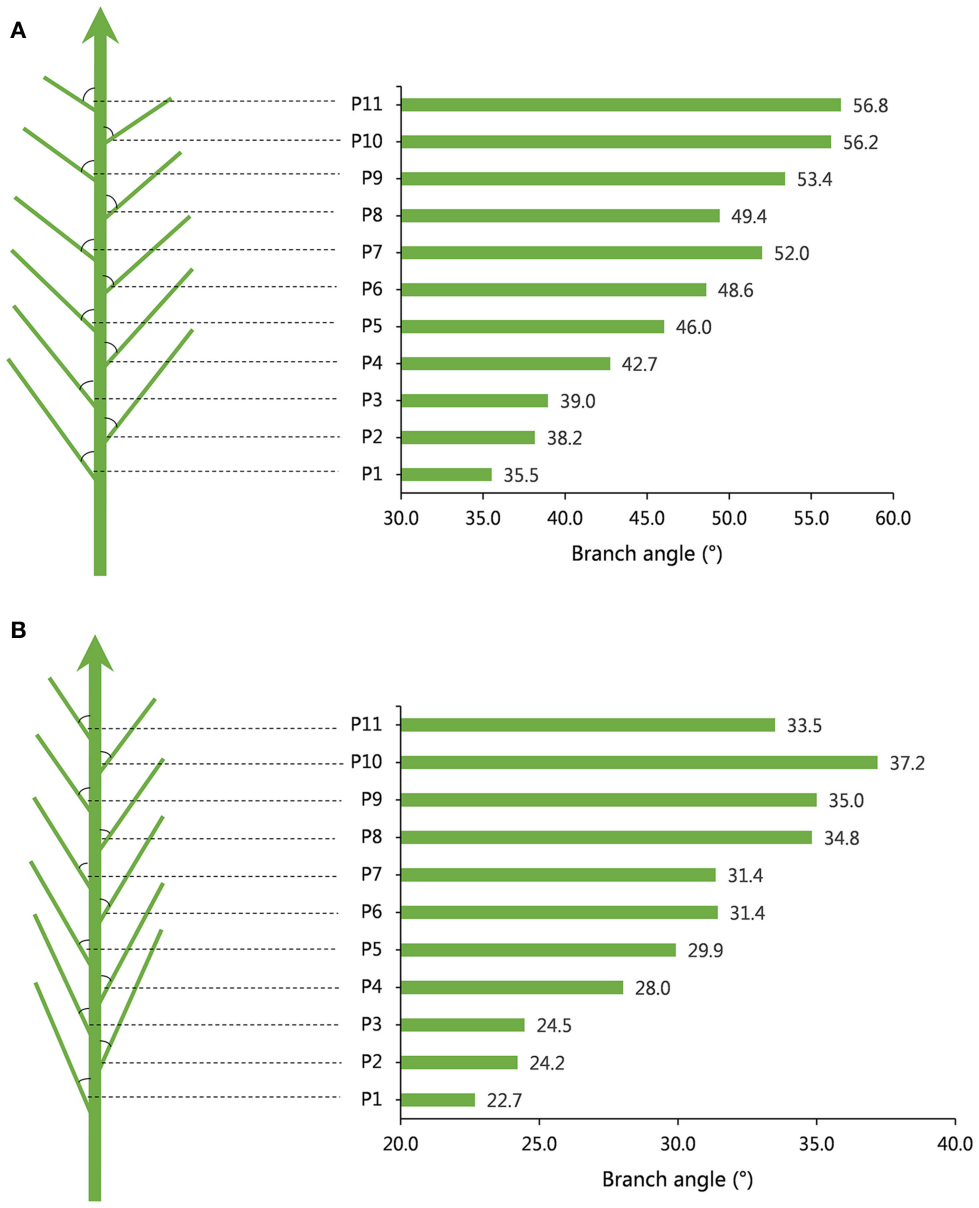
Models of branch angle for loose and compact plant architecture in rapeseed. **(A)** A model for branch angle of loose plant architecture in accession 1218 with a large branch angle and the angle tendency of different branch positions. **(B)** A model for branch angle of compact plant architecture in accession 3304 with a small branch angle and the angle tendency of different branch positions.

The obvious tendency for branch angle was observed in different branch positions of individual plants. Generally, the branch angle would increase with elevating branch physical position regardless of plant architecture (Table 1, Figure 1). For example, in the case of accession 1218, representing loose plant architecture, the angle of the first branch (bottom branch) was 35.52° and the angles of the third, fifth, seventh, ninth and eleventh branch rose gradually to 38.96°, 46.03°, 52.00°, 53.40°, and 56.80°, respectively (Table 1, Figure 1). Another line, 3304, representing compact plant architecture, exhibited a similar branch bending pattern (Table 1, Figure 1). Therefore, the linear growth of branch angle may be affected by the dose-response of auxin concentration. To determine the appropriate measurement region on individual rapeseed plants, a *t*-test of significant differences in different branch regions was conducted. The results demonstrated that there were distinct differences between the upper plant branch angle and the whole plant branch angle in the majority of the rapeseed accessions. The lower plant branch angle showed a similar result, but the middle plant branch angle showed no marked difference compared to the whole plant branch angle (Table S1). For example, in accession 1218, the mean values of the upper, middle, lower and whole plant branch angle were 51.71°, 44.84°, 37.54°, and 44.79°, respectively, and it is evident that the middle plant branch angle was much closer to the whole plant branch angle. Thus, the most representative phenotypic data could be obtained by just measuring the middle branches.

**Table 1:** Branch angles at different positions of individual plants across 40 rapeseed accessions.

**Accession number**	**Branch Angle in different position(°)^a^**												
	**P1^b^**	**P2**	**P3**	**P4**	**P5**	**P6**	**P7**	**P8**	**P9**	**P10**	**P11**	**P12**	**P13**
1192	34.01	36.82	39.20	41.61	42.66	44.88	44.94	46.38	42.16	46.93			
1193	27.76	31.61	33.99	37.91	39.89	42.10	43.83	46.37	43.13	42.20			
1212	34.02	38.81	40.89	42.20	43.49	43.09	44.46	46.47	47.76	43.40			
1216	32.37	35.11	37.34	41.22	44.44	44.31	46.33	49.17					
1218	35.52	38.16	38.96	42.74	46.03	48.60	52.00	49.41	53.40	56.20	56.80		
1241	28.43	28.09	29.51	31.90	33.24	36.90	38.81	39.09	40.78	38.37			
1263	36.70	40.56	41.47	42.78	44.72	41.52	41.74	45.04	45.34	42.40			
1347	23.90	26.36	28.52	28.57	31.92	32.34	33.28						
2692	30.64	36.33	36.07	40.62	42.87	43.83	46.37	46.64					
2738	29.98	31.78	31.50	35.79	37.84	40.28	43.13	41.95	45.50	46.73	45.50	56.10	
2787	33.58	35.87	38.21	41.08	44.19	45.95	44.40	50.43	44.70				
2874	25.38	25.98	26.64	26.87	28.82	30.63	30.00	30.39	32.56	34.90	38.63	33.15	29.65
2893	25.33	26.64	27.99	29.14	32.60	34.23	33.82	29.40					
3078	44.71	46.74	48.33	51.35	54.47	54.20	52.18	53.23	52.50				
3100	31.84	34.02	40.14	43.40	44.96	45.39	46.85	47.38	48.50				
3112	23.78	24.09	25.21	26.66	31.49	31.06	31.02	31.02	36.83	37.70			
3260	25.81	25.78	28.09	29.74	31.94	33.51	33.29	35.09	35.54	35.10	41.20		
3264	24.04	23.78	25.08	28.94	30.36	33.09	34.26	34.70	33.73	34.30	33.40		
3279	29.59	29.49	31.92	33.14	33.90	36.28	37.73	39.91	36.82	38.80	40.40	42.50	43.75
3298	25.36	25.28	27.46	28.90	29.81	32.98	35.78	33.93	37.64	37.43	43.65	43.90	
3304	22.68	24.21	24.47	28.02	29.92	31.43	31.36	34.83	35.00	37.20	33.50		
3307	24.88	27.31	30.10	32.44	34.10	36.20	39.32	39.02					
3362	24.93	27.36	29.13	29.41	31.29	33.46	35.03	32.26	35.25	32.93	38.25		
3367	26.31	26.42	28.39	30.63	33.32	34.86	32.30	35.34					
3386	26.70	28.32	31.17	32.69	35.40	35.72	35.47	32.97	33.40				
3399	25.63	26.08	27.30	28.22	29.84	34.46	34.10	35.80					
3401	39.71	41.48	43.42	45.33	47.98	45.83	46.54	53.55					
3443	24.23	26.79	28.99	31.47	34.63	35.73	36.20	40.44	40.95				
3453	26.97	28.38	29.68	32.78	35.61	37.58	37.80	40.30					
3458	26.90	27.53	28.41	30.73	33.26	32.76	33.33	33.10	32.20	34.85	36.40		
3477	23.76	25.51	27.41	29.10	29.20	32.51	31.98	33.35	33.57				
3480	23.81	25.51	26.97	29.57	30.43	34.02	34.62	36.88					
3501	26.22	27.06	29.72	31.34	34.32	36.16	37.05	34.60	39.30				
4604	30.82	32.69	36.50	40.07	40.77	41.96	43.89	45.04	43.85	34.05			
4625	42.61	45.28	47.96	46.52	46.35	49.82	45.35						
4679	29.93	32.09	35.45	38.27	38.21	38.78	34.75						
4712	26.74	30.04	31.92	35.98	39.62	42.13	43.83	46.60					
5102	31.28	34.53	38.08	41.02	44.87	46.99	47.79	46.59	46.30	49.35			
5160	32.42	34.51	36.02	38.63	39.95	39.81	41.11	42.05	44.40				
6146	26.98	27.19	29.84	30.72	32.20	32.78	35.84	35.72	36.90				

a *The branch angles were measured from bottom to top of individual plant on E2*.

b *P1: The first position, the rest, and so on*.

### Phenotypic variation of branch angle in an association mapping population

**Figure 2:**
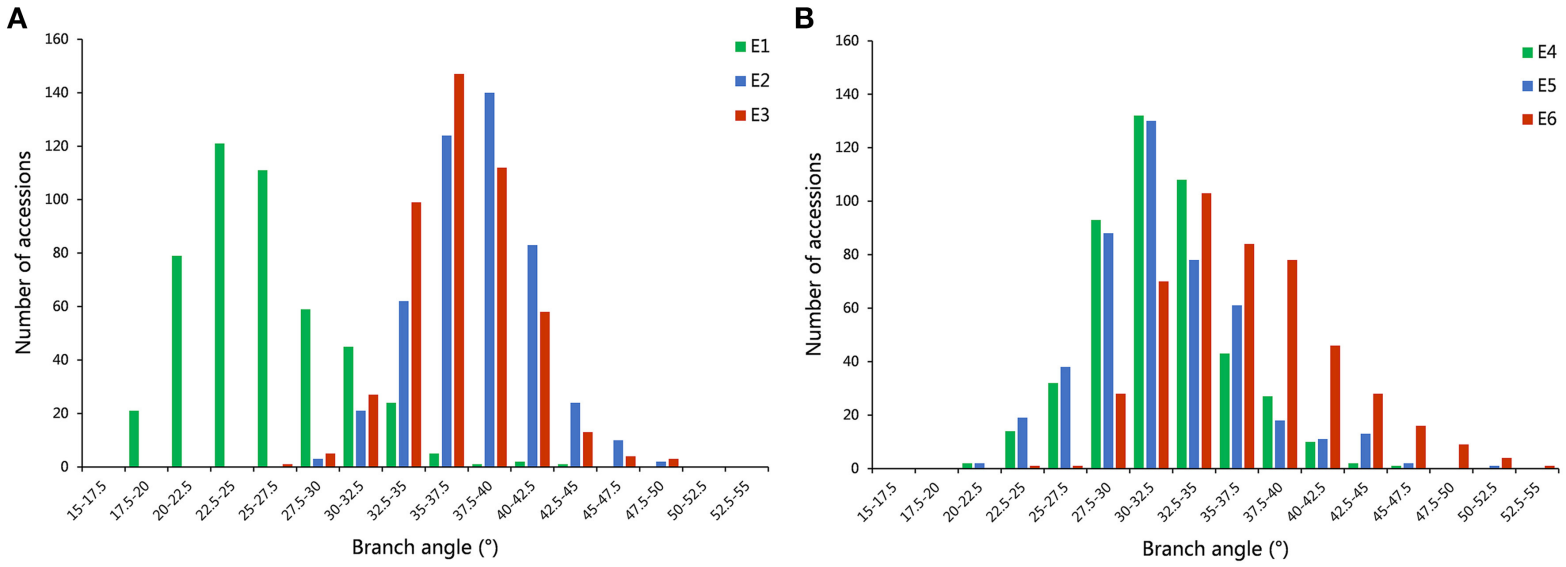
The distribution of branch angle across 472 rapeseed accessions in six environments. **(A)** The environments of E1, E2, and E3. **(B)** The environments of E4, E5, and E6.

A distinct phenotype variation in branch angle, ranging from 17.5° to 53.6°, was found across the 472 rapeseed accessions in the six environments (Figure 2, Table 2). The maxima in the observed phenotype data were 1.8–2.5 times the minima, varying from 17.5° to 43.9° in E1, 28.0° to 49.3° in E2, 26.4° to 48.3° in E3, 20.6° to 46.9° in E4, 22.1° to 50.4° in E5 and 24.1° to 53.6° in E6. Moreover, two adjacent locations, Wuhan and Yangluo, exhibited analogous phenotypic variation both in 2015 (E2 and E3) and in 2016 (E4 and E5), indicating that the branch angle is a relative stably inherited trait (Table 2). The correlation coefficient of branch angle, ranging from 0.49 to 0.64, indicated that the branch angles in six environments have a significantly positive correlation (*p* < 0.05, Table 3).The broad-sense heritability (*H*^2^) of branch angle among the rapeseed panel was 76.06% (Table S2), suggesting that environmental factors had limited influence on the branch angle, which exhibited a fairly stable manner.

**Table 2:** Phenotypic variation in branch angle for a rapeseed association population in six environments.

**Environments**	**Min (°)**	**Max (°)**	**Mean ±SD (°)**	**CV (%)**
E1	17.5	43.9	25.9 ± 4.1	15.9
E2	28.0	49.3	38.0 ± 3.4	8.9
E3	26.4	48.3	36.9 ± 3.2	8.8
E4	20.6	46.9	31.9 ± 3.8	12.0
E5	22.1	50.4	32.2 ± 4.5	13.8
E6	24.1	53.6	36.6 ± 4.9	13.4

*SD, standard deviation; CV, coefficient of variation*.

**Table 3:** Correlation analysis of branch angle between environments.

**Environments**	**E1**	**E2**	**E3**	**E4**	**E5**
E2	0.57^*^				
E3	0.53^**^	0.59^**^			
E4	0.54^**^	0.57^**^	0.54^**^		
E5	0.49^**^	0.58^**^	0.54^**^	0.64^**^	
E6	0.54^**^	0.60^**^	0.54^**^	0.57^**^	0.58^**^

*, ** *Significant difference at the 5 and 1% level, respectively*.

### Haplotype block structure study

**Figure 3:**
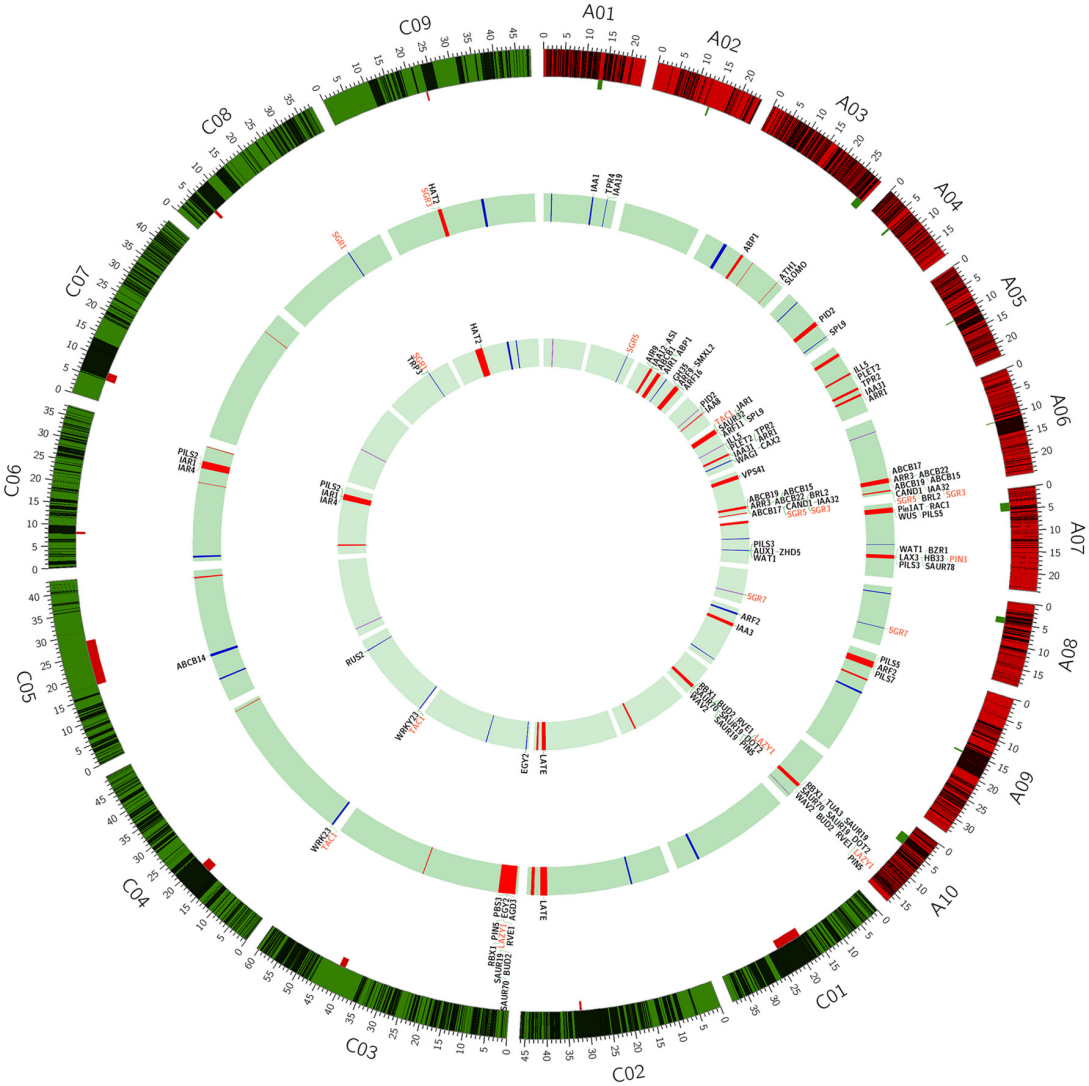
Mapping of haplotype blocks and association loci for branch angle on the *Brassica napus* genome. The black bands in the outer circle indicate the haplotype blocks; the location, and size of centromeres are marked by the rectangles attached to the outer circle. The bands in the middle and inner circle indicate the association loci for branch angle that are identified in the MLM and MRMLM model, respectively. Three types of loci (QTL, LD block, and no block) are colored in red, blue and violet, respectively. The identified genes are listed on the outside of the middle and inner circles, and the genes of interest are colored in red.

**Figure 4:**
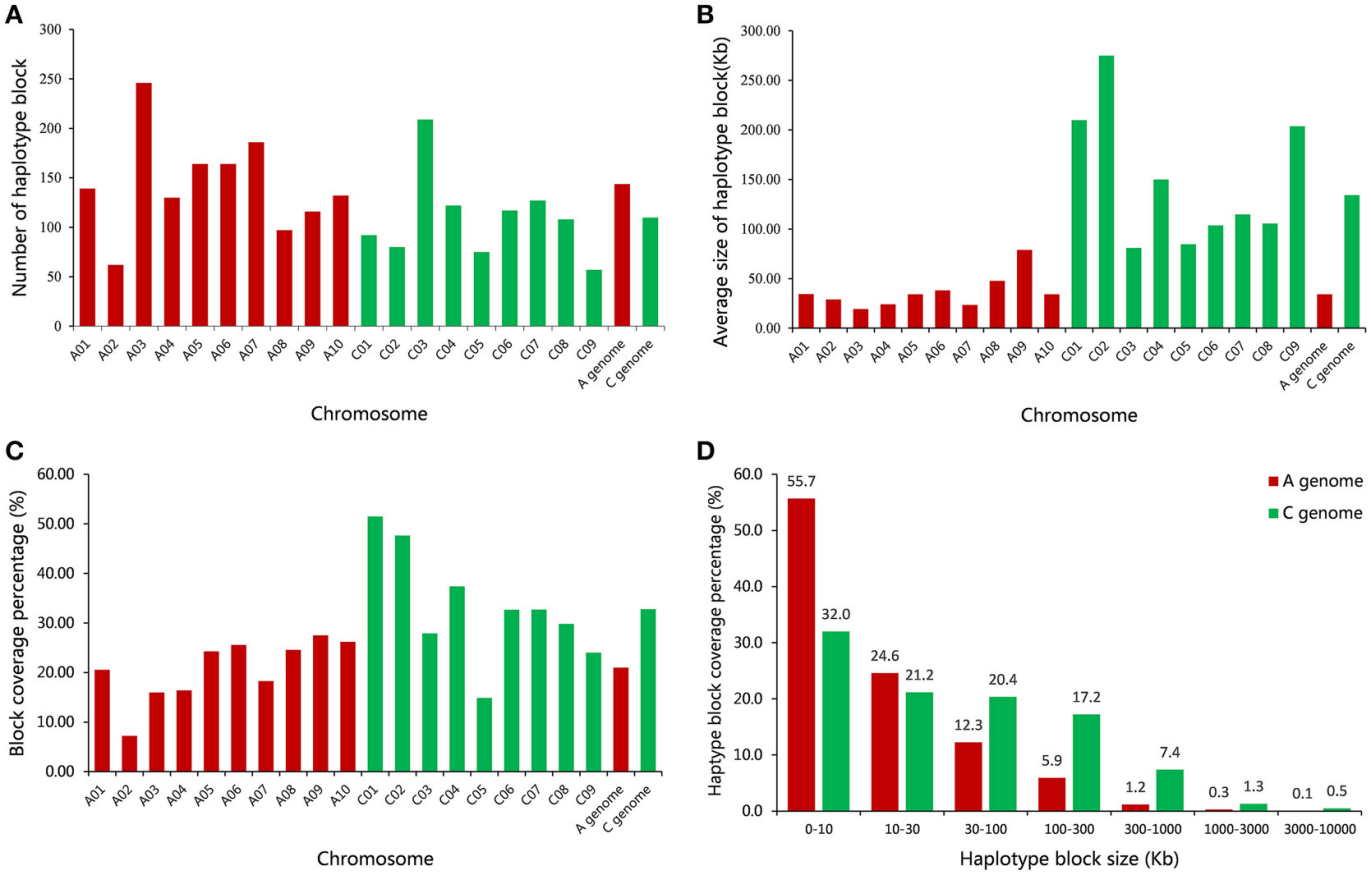
Comparative analysis of haplotype block structure in the A-subgenome and C-subgenome of rapeseed. **(A)** A comparison of the number of haplotype blocks on 19 rapeseed chromosomes. **(B)** A comparison of the average size of haplotype blocks on 19 rapeseed chromosomes. **(C)** A comparison of the haplotype block coverage percentage on 19 rapeseed chromosomes. **(D)** A comparison of the haplotype block size range distributions on the A- and C-subgenome.

A total of 2,423 conserved haplotype blocks were detected after estimating 19,945 high-quality SNPs distributing on the whole-genome in 472 rapeseed accessions, which spanned 181.53 Mb and covered 28.28% of the assembled *B*. *napus* genome (Figure 3, Table S3). The haplotype block position, length, and SNP number within each block is also provided in Table S3. The average number of haplotype blocks in the A-subgenome chromosomes was 143.6 (ranging from 62 to 246) with an average block size 34.10 kb (ranging from 19.30 to 78.97 kb), and a haplotype block coverage percentage ranging from 7.23 to 27.52%, with a mean percentage of 21.00% (Table 4, Figures 4A–C). In the C-subgenome chromosomes, the haplotype block number varied from 57 to 209 (average number of 109.7) with a fairly larger haplotype block size ranging from 80.86 to 274.85 kb (average size 134.30 Kb), and with a block coverage proportion varing from 14.87 to 51.49% (average proportion of 32.80%, Table 4, Figures 4A–C). In the A-subgenome, haplotype blocks less than 30 kb in size were roughly four-fifths of all blocks (80.3%), and, this proportion was more than one-half (53.2%) in the C-subgenome (Figure 4D).

**Table 4:** Summary of haplotype block structure across 472 rapeseed accessions.

**Chromosome**	**Number of blocks**	**Total block size (Kb)**	**Mean block size (Kb)**	**Percentage of block coverage on chromosome (%)**
A01	139	4,773.34	34.34	20.54
A02	62	1,792.58	28.91	7.23
A03	246	4,748.94	19.30	15.97
A04	130	3,131.54	24.09	16.39
A05	164	5,588.16	34.07	24.27
A06	164	6,244.79	38.08	25.61
A07	186	4,379.38	23.55	18.29
A08	97	4,632.21	47.75	24.56
A09	116	9,161.08	78.97	27.52
A10	132	4,515.45	34.21	26.19
C01	92	19,296.25	209.74	51.49
C02	80	21,988.37	274.85	47.66
C03	209	16,900.74	80.86	27.91
C04	122	18,285.34	149.88	37.40
C05	75	6,341.23	84.55	14.87
C06	117	12,132.43	103.70	32.67
C07	127	14,581.93	114.82	32.69
C08	108	11,417.34	105.72	29.81
C09	57	11,614.30	203.76	24.00
A mean	143.60	4,896.75	34.10	21.00
C mean	109.67	14,728.67	134.30	32.80

There were 23 haplotype blocks whose size was more than 1 Mb, accumulatively accounting for more than one-third of the total block size (Table 5). These blocks were distributed on 12 rapeseed chromosomes and most of them (18/23) were located on the C-subgenome (Table 5). Approximately half of the blocks (11/23) were across or in the vicinity of their corresponding centromere (Table 5 and Figure 3, Mason et al., 2016), meaning that stronger LD existed in these blocks; furthermore, if the SNPs located on the blocks are excluded, the LD decay will depress sharply (Qian et al., 2014; Sun et al., 2016b).

**Table 5:** The large haplotype block (≥1 Mb) and the corresponding centromere on rapeseed chromosome.

**Chromosome**	**Haplotype block**			**Centromere**		
	**Start (Mb)**	**End (Mb)**	**Length (Mb)**	**Start (Mb)**	**End (Mb)**	**Length (Mb)**
A05^*^	12.4	13.6	1.2	10.9	10.9	0.07
A06	11.1	14.5	3.3	11.1	11.1	0.02
A09	14.2	15.7	1.5	15.6	15.9	0.3
A09	17.3	19.3	2.0			
A10	3.1	4.5	1.4	2.9	5.3	2.4
C01	17.9	24.7	6.8	17.9	24.2	6.2
C01	24.7	26.2	1.4			
C01	27.9	29.4	1.4			
C02	23.3	25.2	1.9			
C02	26.5	27.5	1.0			
C02	27.6	33.6	6.0	31.8	32.2	0.3
C03	52.0	53.4	1.5			
C03	54.6	55.7	1.1			
C04	15.3	21.7	6.4	17.1	19.4	2.3
C04	22.1	23.3	1.1			
C06	7.9	9.9	1.9	8.0	8.4	0.5
C07	5.6	11.3	5.7	5.4	7.2	1.8
C07	11.4	13.5	2.1			
C08	4.1	5.7	1.6			
C08	6.4	9.5	3.1	5.8	6.4	0.6
C09	11.0	12.5	1.5			
C09	23.5	26.1	2.6	23.1	23.4	0.3
C09	37.6	39.1	1.5			

* *12.40–14.12 Mb on A5 chromosome of reference genome (Darmor v4.1) in wrong place, should be near centromere at 10.86 Mb (Mason et al., 2016)*.

### Genome-wide association analysis

**Figure 5:**
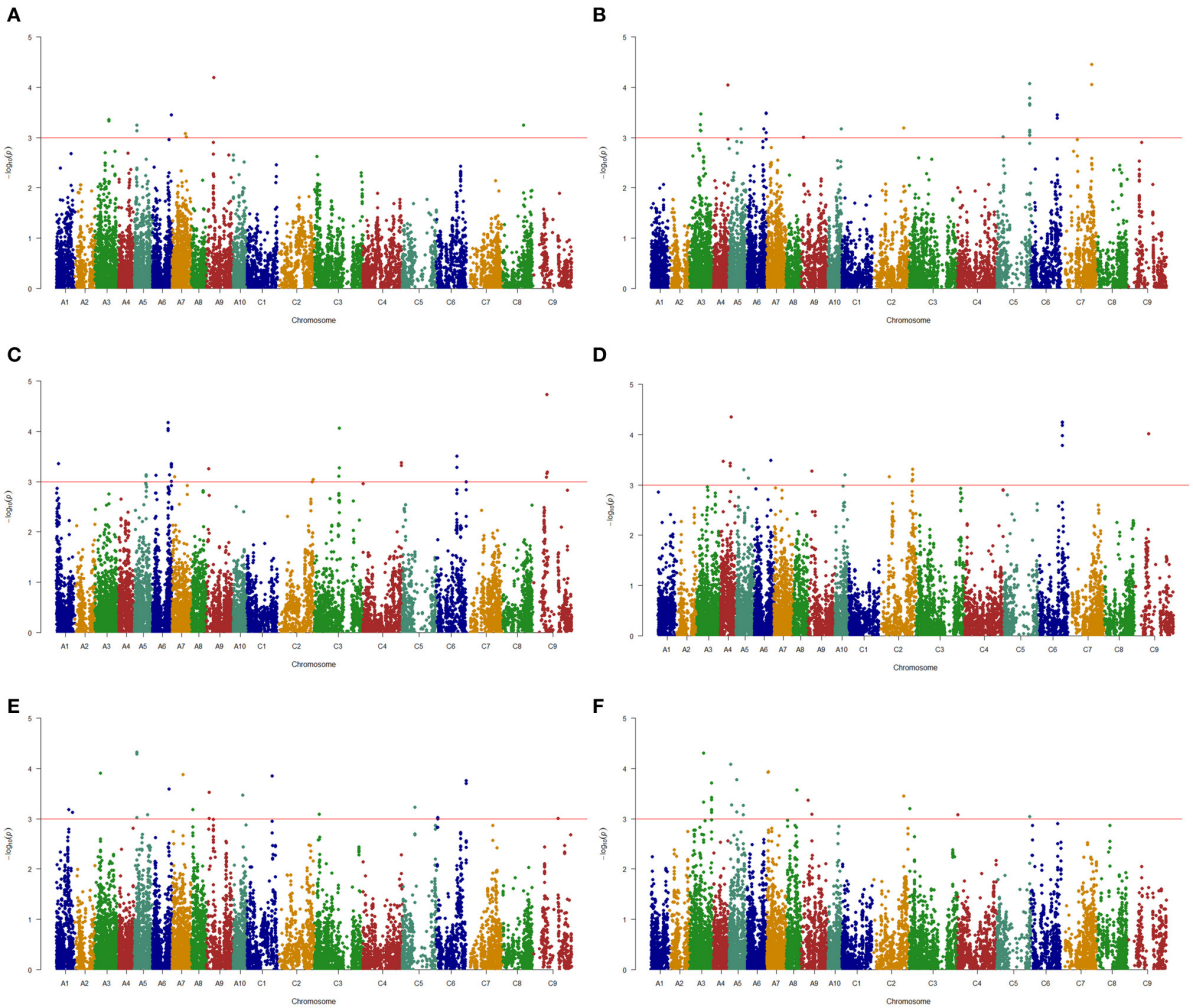
Manhattan plots of association analysis for branch angle using the Q+K model in six environments. **(A)** E1. **(B)** E2. **(C)** E3. **(D)** E4. **(E)** E5. **(F)** E6. The horizontal red line indicates the significance threshold [–log_10_ (*p*) = 3.0].

A total of 144 and 69 significantly-associated SNPs of branch angle were detected, which could explain up to 62.2 and 66.2% of the cumulative phenotypic variation, using the BLUP value and individual environment in MLM and MRMLM, respectively (Figure 5, Table S4). The significantly-associated SNPs corresponded to 46 and 38 loci in MLM and MRMLM, respectively (Figure 3, Table S5), and 21 loci among them partly shared the region between the two models, which accounted for 45.65% (21/46) and 55.26% (21/38) of the total identified loci (Table 6). For instance, two vicinity loci on A6 were both repeatedly detected in the two models. In MLM, the first locus on A6 was crossed from 19,416,065 to 20,815,553 with a peak SNP (highest significant) of Bn-A06-p18028879, which contributed to 4.14% of the phenotypic variance. In comparison, the homologous locus on A6 in MRMLM spanned from 19,630,281 to 20,815,553, with a peak SNP Bn-A06-p18246821, which explained 5.01% of the phenotypic variance. The second locus on A6 in MLM was crossed from 23,211,156 to 23,499,018, with a peak SNP Bn-A06-p24551529 which contributed to 3.53% of the phenotypic variance. In MRMLM, the corresponding locus on A6 spanned from 23,362,162 to 23,495,968, with a peak SNP Bn-A06-p24544753, which explained 3.75% of the phenotypic variance. In a word, the two loci on A6 in MLM shared 84.7 and 46.5% of their regions with the homologous loci in MRMLM (Table 6).

**Table 6:** The common loci significantly associated with branch angle between MLM and MRMLM.

**Peak SNP**	**Chr.^c^**	**Position (bp)**	**Locus range (bp)**	**−log_10_(P)**	***R*^2^ (%)**	**Environments**
Bn-A03-p7631083^a^	A03	6,932,172	6,443,943–7,409,012	3.90	4.47	E5
Bn-A03-p7631083^b^	A03	6,932,172	6,932,172–8,182,258	7.01	7.38	E2,E5,E6,BLUP
Bn-A03-p14462148^a^	A03	13,631,937	12,893,594–1,3631,937	3.47	3.59	E2,BLUP
Bn-A03-p12359936^b^	A03	11,441,915	11,441,915–1,3478,452	3.17	2.62	E2,E4
Bn-A03-p18455020^a^	A03	17,448,094	17,448,094–17,470,198	4.30	4.35	E1,E6,BLUP
Bn-A03-p18455020^b^	A03	17,448,094	17,448,094–17,529,460	6.13	5.23	E3
Bn-A04-p12372810^a^	A04	13,355,441	12,032,695–1,3355,441	4.35	4.41	E2,E4
Bn-A04-p10541268^b^	A04	11,685,599	11,685,599–12,075,741	5.26	4.81	E3,E4
Bn-A05-p3135213^a^	A05	3,161,291	2,335,746–3,301,547	4.32	4.42	E1,E5,E6,BLUP
Bn-A05-p2208960^b^	A05	2,335,746	568,294–3,155,611	5.97	4.35	E2,E5,E6
Bn-A05-p16260894^a^	A05	14,976,591	14,807,389–15,574,266	3.42	3.77	E2,E3,E4,BLUP
Bn-A05-p16844504^b^	A05	15,534,495	15,534,495–16,389,270	5.61	4.48	E1,E2,BLUP
Bn-A06-p4316905^a^	A06	4,115,493	4,065,493–4,165,493	3.13	3.09	E3
Bn-A06-p3587032^b^	A06	3,371,354	2,448,863–4,414,959	5.00	6.32	E1,E5,E6,BLUP
Bn-A06-p18028879^a^	A06	19,416,065	19,416,065–20,815,553	4.17	4.14	E2,E3,E4,E5,BLUP
Bn-A06-p18246821^b^	A06	19,630,281	19,630,281–20,815,553	5.38	5.01	E1,E2,E3,E5,E6,BLUP
Bn-A06-p24551529^a^	A06	23,499,018	23,211,156–23,499,018	3.49	3.53	E1,E2,E3
Bn-A06-p24544753^b^	A06	23,495,968	23,362,162–23,495,968	5.13	3.75	E2,E3,BLUP
Bn-A07-p14798978^a^	A07	16,678,307	16,678,307–17,777,339	3.08	3.08	E1
Bn-A07-p14798978^b^	A07	16,678,307	16,644,403–16,696,772	4.70	4.81	E1
Bn-scaff_17174_1-p388642^a^	A09	89,852,95	8,505,452–8,985,295	4.19	4.78	E1,E6,BLUP
Bn-scaff_17174_1-p388642^b^	A09	89,852,95	8,505,452–10,091,847	4.15	6.05	E1,BLUP
Bn-A10-p10252741^a^	A10	11,639,365	11,639,365–12,662,334	3.47	3.53	E4,E5,BLUP
Bn-A10-p13243690^b^	A10	13,273,350	11,639,365–1,3273,350	5.24	6.13	E1,E5,E6,BLUP
Bn-scaff_15879_1-p79732^a^	C01	31,397,780	31,280,411–31,792,393	3.85	4.01	E5
Bn-scaff_15879_1-p79732^b^	C01	31,397,780	30,661,223–31,397,780	3.92	5.63	E5
Bn-scaff_22144_1-p193415^a^	C02	39,783,194	39,783,194–41,853,453	3.45	3.72	E2,E4,E6,BLUP
Bn-scaff_22144_1-p193415^b^	C02	39,783,194	39,783,194–41,769,858	4.08	5.27	E2,E4,E5,E6
Bn-scaff_17177_1-p365264^a^	C02	44,856,206	43,919,691–44,856,206	3.04	3.03	E3
Bn-scaff_17721_1-p381227^b^	C02	43,919,691	43,919,691–44,656,670	5.36	5.87	E3,E6
Bn-scaff_16614_1-p1291979^a^	C03	830,111	830,111–6,135,024	3.20	3.50	E5,E6
Bn-scaff_16614_1-p722822^b^	C03	1,380,050	1,241,778–1,381,475	3.61	2.39	E1
Bn-scaff_16935_1-p98166^a^	C04	482,636	90,141–483,903	3.08	3.09	E6
Bn-scaff_16935_1-p98166^b^	C04	482,636	90,141–483,903	4.46	4.16	E5,E6,BLUP
Bn-scaff_18807_1-p726783^a^	C06	30,124,980	30,013,719–32,253,524	4.24	4.47	E2,E4,BLUP
Bn-scaff_23821_1-p45657^b^	C06	30,013,719	30,013,719–32,994,356	3.23	3.50	E4,E6
Bn-scaff_16770_1-p4296727^a^	C08	26,985,890	26,949,751–27,107,526	3.25	3.83	E1
Bn-scaff_16770_1-p4296727^b^	C08	26,985,890	26,949,751–26,985,890	3.12	3.99	E1
Bn-scaff_15650_1-p624000^a^	C09	17,390,383	17,284,551–18,368,456	4.72	4.71	E3,E4,BLUP
Bn-scaff_15650_1-p624000^b^	C09	17,390,383	13,551,211–17,390,383	4.61	4.65	E2,E3,BLUP
Bn-scaff_20619_1-p159276^a^	C09	31,686,981	31,412,461–32,095,073	3.00	3.05	E5
Bn-scaff_20619_1-p159276^b^	C09	31,686,981	31,412,461–32,162,770	4.52	4.52	E5

a *The locus was detected in MLM*.

b *The locus was detected in MRMLM*.

c *Chromosome*.

Furthermore, 45.65% (21/46) and 52.63% (20/38) of the identified loci in MLM and MRMLM were verified in at least two environments, illustrating that our association results were credible and reproducible (Table S5). Furthermore, these loci were distributed on all the chromosomes in both models except for A2 in MLM (Figure 3, Table S5). Approximately three-fifths (27/46) of the loci were located on the A sub-genome in MLM, and the average number of loci in each chromosome was 2.5 (ranging from 1 to 4; Figure 3, Table S5). In MRMLM, a similar proportion of loci (23/38) were located on the A sub-genome; the average number of loci in each chromosome was 2.0 (ranging from 1 to 4; Figure 3, Table S5).

### Candidate genes identification

Using the *A. thaliana* orthologous genes and published literatures about branch angle as a reference, altogether 73 and 65 candidate genes, corresponding to 32 and 28 loci, were identified in MLM and MRMLM, and 43 candidate genes were commonly detected in the two models (Figure 3, Table S5).

*LAZY1* and *TAC1* are well-known genes modulating branch angle, whose mutants display converse branch angle morphology in plants (Yu et al., 2007; Yoshihara et al., 2013). We identified two orthologs of *LAZY1, BnaA10g19550D* and *BnaC03g06250D*, at 13.9 Mb on A10 and 3 Mb on C3, which are 2,213 kb and 2,202 kb from the peak SNPs of Bn-A10-p10252741 and Bn-scaff_16614_1-p1291979, respectively (Table 7, Table S5A). Furthermore, the *TAC1* ortholog, *BnaC04g00780D*, was detected at 0.7 Mb on C4, which is 183 kb from the peak SNP Bn-scaff_16935_1-p98166 (Table 7, Table S5A).

**Table 7:** Summary of candidate genes of interest.

**Candidate gene**	**Ortholog**	**Chr^a^**	**Position**	**MLM**		**MRMLM**	
				**Peak SNP**	**Distance from candidate gene (Kb)^b^**	**Peak SNP**	**Distance from candidate gene (Kb)^b^**
*SGR1*	*BnaC08g25070D*	C8	26,942,422	Bn-scaff_16770_1-p4296727	43 (Downstream)	Bn-scaff_16770_1-p4296727	43 (Downstream)
*SGR3*	*BnaA06g35880D*	A6	23,503,576	Bn-A06-p24551529	5 (Upstream)	Bn-A06-p24544753	8 (Upstream)
	*BnaC09g19750D*	C9	16,839,283	Bn-scaff_15650_1-p624000	551 (Downstream)		
*SGR5*	*BnaA06g34390D*	A6	22,719,636	Bn-A06-p24551529	779 (Downstream)	Bn-A06-p24544753	776 (Downstream)
	*BnaA02g26100D*	A2	19,178,238			Bn-A02-p22843446	1311 (Downstream)
*SGR7*	*BnaA08g15740D*	A8	13,083,464	Bn-A08-p15792942	205 (Downstream)	Bn-A08-p16403550	787 (Downstream)
*LAZY1*	*BnaA10g19550D*	A10	13,852,425	Bn-A10-p10252741	2,213 (Upstream)	Bn-A10-p13243690	579 (Upstream)
	*BnaC03g06250D*	C3	3,032,284	Bn-scaff_16614_1-p1291979	2,202 (Upstream)		
*TAC1*	*BnaA05g01220D*	A5	720,556			Bn-A05-p2208960	1,615 (Downstream)
	*BnaC04g00780D*	C4	665,502	Bn-scaff_16935_1-p98166	183 (Upstream)	Bn-scaff_16935_1-p98166	183 (Upstream)
*PIN3*	*BnaA07g23670D*	A7	17,763,973	Bn-A07-p14798978	1,085 (Upstream)		

a *Chromosome*.

b *The distance of SNP and its upstream or downstream from candidate gene*.

**Figure 6:**
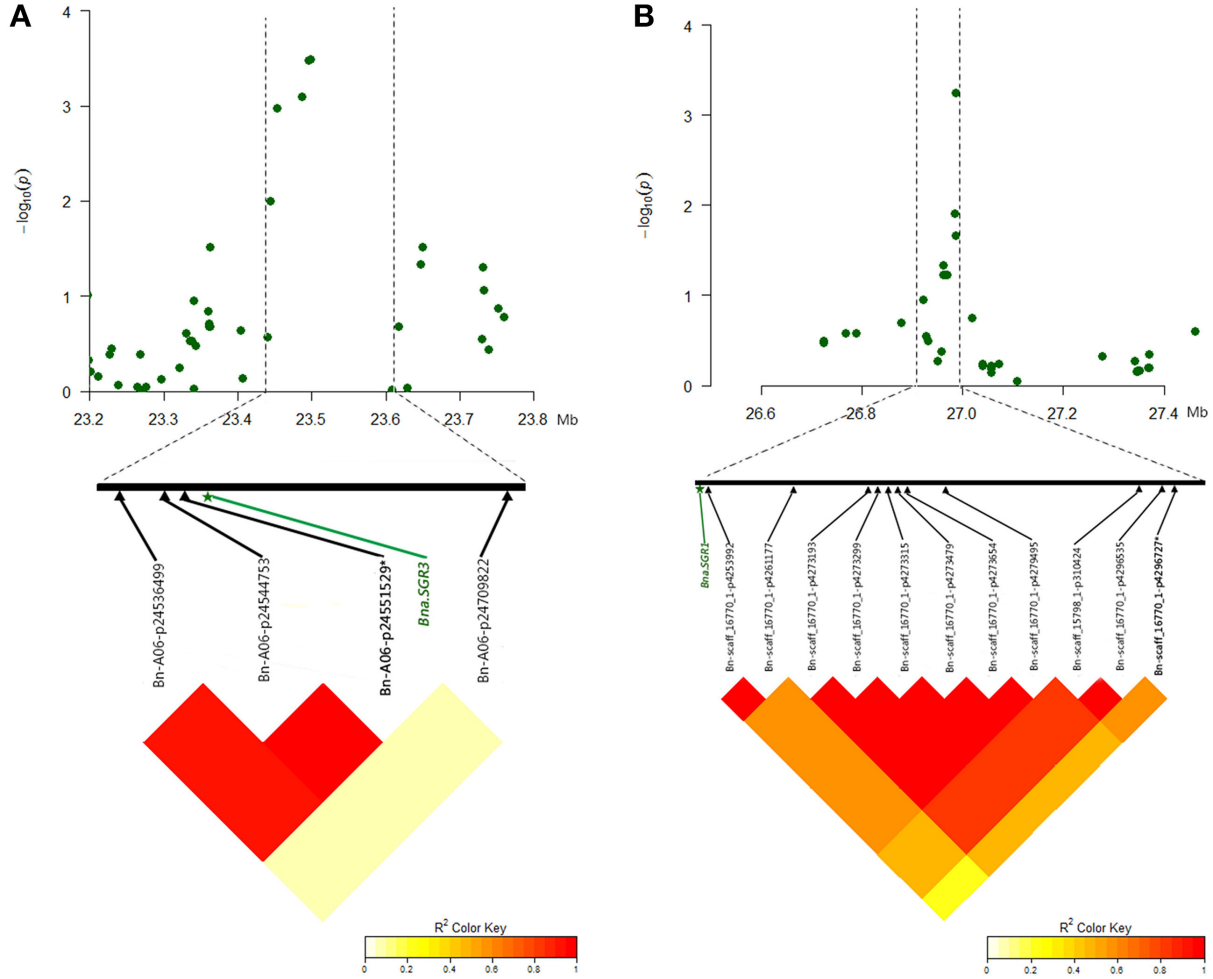
Significantly associated SNPs and corresponding candidate genes identified for branch angle in MLM. **(A)** Manhattan plot of the A6 chromosomal region around the candidate *BnaA06g35880D* (*Bna.SGR3*) in E2 and LD plot with a peak SNP Bn-A06-p24551529 and both flanking SNPs. **(B)** Manhattan plot of C8 chromosomal region around candidate *BnaC08g25070D* (*Bna.SGR1*) in E1 and LD plot with a peak SNP Bn-scaff_16770_1-p4296727 and both flanking SNPs. The SNP written in bold and marked with an asterisk indicates the peak SNP.

**Figure 7:**
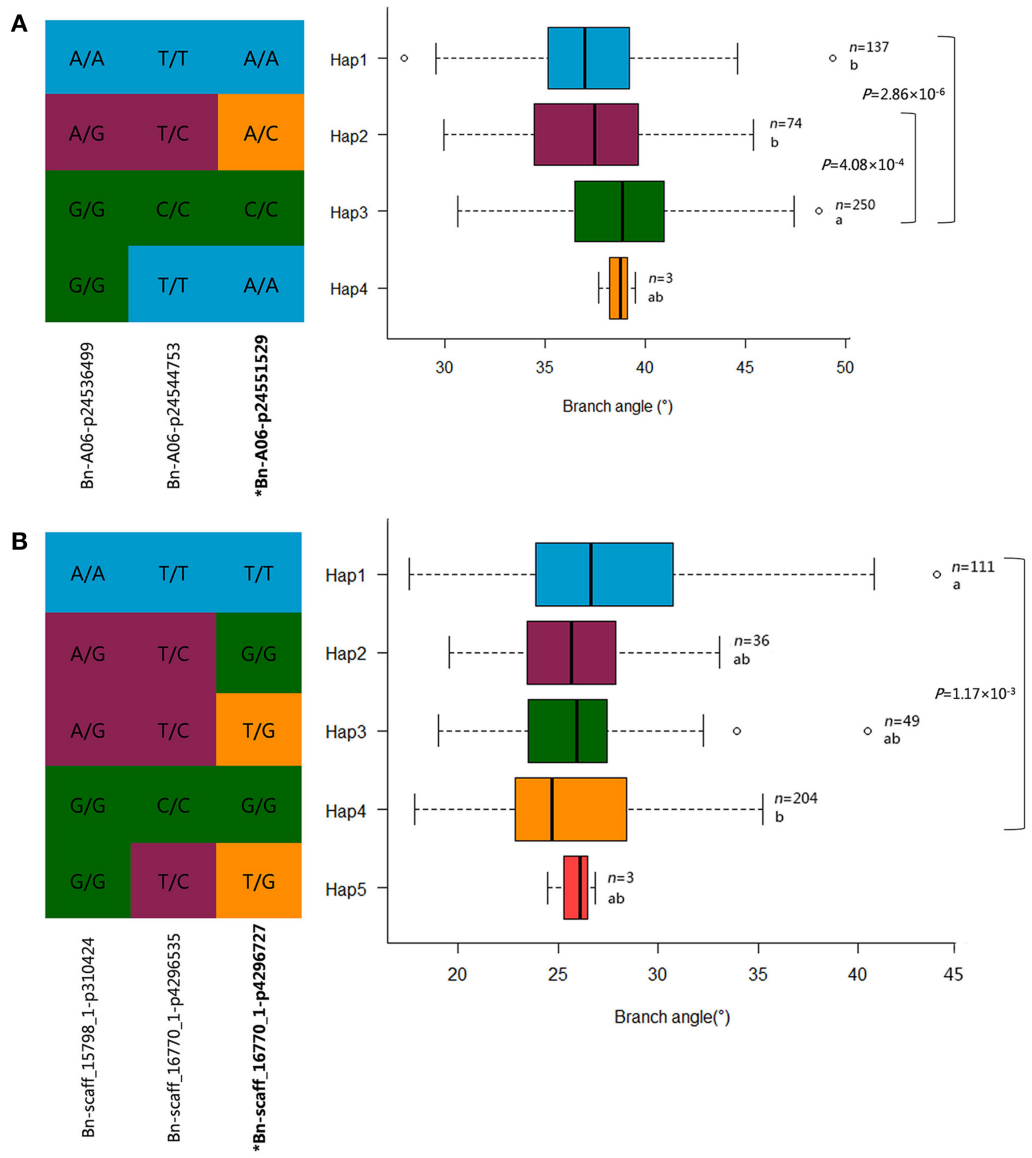
Haplotypes of associated SNPs among rapeseed natural variations. **(A)** Haplotype analysis of the peak SNP Bn-A06-p24551529 for *Bna.SGR3* in MLM. **(B)** Haplotype analysis of the peak SNP Bn-scaff_16770_1-p4296727 for *Bna.SGR1* in MLM. *n* denotes the number of genotypes belonging to each haplotype group, and the genotypes less than three are not shown. Statistical significance was determined with a *t*-test, different letters represent significant a difference at 5% level. The branch angle distribution of each haplotype group is displayed with a box plot.

The mutants of *sgr1*–*sgr7* showed a defective in gravitropic response resulting in the alteration of normal branch angle in *Arabidopsis* (Fukaki et al., 1996; Yamauchi et al., 1997). Two orthologs of *SGR3* in rapeseed, *BnaA06g35880D* and *BnaC09g19750D*, were identified at 23.5 Mb on A6 and 16.8 Mb on C9, which were 5 kb from the peak SNP Bn-A06-p24551529 and 551 kb away from the peak SNP Bn-scaff_15650_1-p624000, respectively (Table 7, Figure 6A, Table S5A).The haplotype of the peak SNP Bn-A06-p24551529 for *SGR3* was analyzed, and 472 rapeseed genotypes were classified into four haplotype groups (Figure 7A). Haplotype 3 (Hap3, *n* = 250) was the largest group, Hap1 (*n* = 137) and Hap3 (*n* = 74) were the second and third largest group, and Hap4 (*n* = 3) was a minor group comprising a few rapeseed lines. Statistically, accessions with Hap1 and Hap2 had a significantly lower branch angle than those with Hap3 (*P* = 2.86 × 10^−6^ and 4.08 × 10^−4^, respectively, Figure 7A).

The ortholog of *SGR1, BnaC08g25070D*, was located at 26.9 Mb on C8, which is 43 kb from the peak SNP Bn-scaff_16770_1-p4296727 (Table 7, Figure 6B, Table S5A). The results of the haplotype effect analysis for Bn-scaff_16770_1-p4296727 illustrates that the average branch angle of individuals with the Hap1 allele was prominently lower than that with Hap4 (*P* = 1.17 × 10^−3^, Figure 7B). The *SGR5* ortholog *BnaA06g34390D* was detected at 22.7 Mb on A6, which is 779 kb upper from the peak SNP Bn-A06-p24551529, which was shared with *SGR3* (Table S5A). In addition, the ortholog of *SGR7, BnaA08g15740D*, was characterized at 13.1 Mb on A8, which is 205 kb from the SNP Bn-A08-p15792942 (Table 7, Table S5A).

The branch curvature growth results from auxin asymmetry accumulation between the upper and bottom portion of this organ; the genes involved in auxin homeostasis are required in this process (Roychoudhry and Kepinski, 2015). This category gene was also characterized in our study, for example, the ortholog of *PIN3 BnaA07g23670D*, the member of the auxin efflux carrier family, was detected at 17.8 Mb on A7, which is 1,085 kb down from the peak SNP Bn-A07-p14798978 (Table 7, Table S5A). More information about the other genes identified in the present study for branch angle are available in the Table S5A; the above-mentioned orthologs in rapeseed were derived from the MLM, and the homologous genes from MRMLM are listed in Table S5B.

### Candidate genes validation

**Figure 8:**
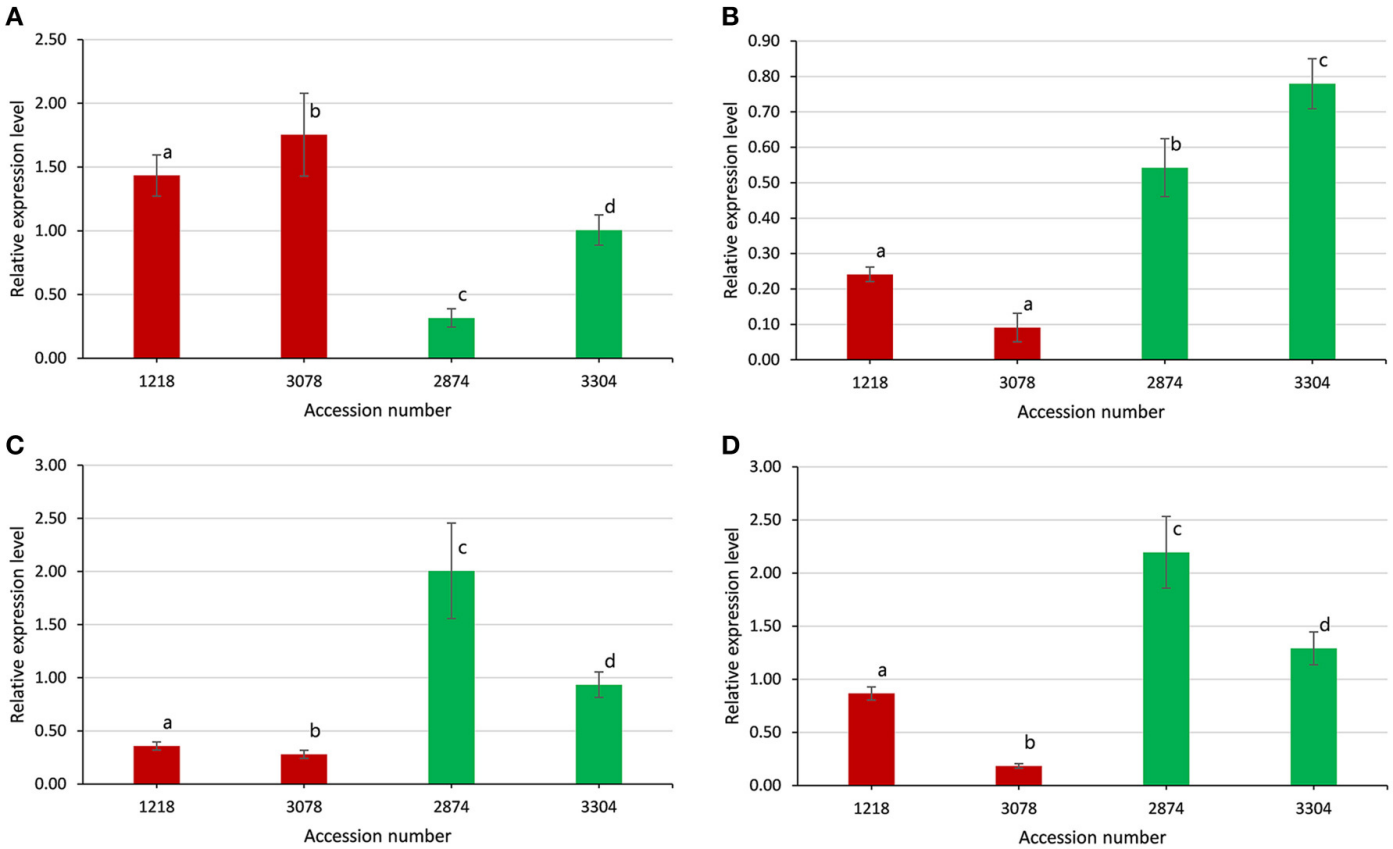
Expression levels of four candidate genes between extremely large and small branch angle accessions. **(A)** *Bna*.*TAC1*. **(B)** *Bna*.*SGR1*. **(C)** *Bna*.*SGR3*. **(D)** *Bna*.*SGR5*. Error bars, s.d.; statistical significance was determined with a *t*-test, different letters above the bar represent significant a difference at 5% level.

Four identified candidate genes, i.e., *TAC1, SGR1, SGR3*, and *SGR5*, were selected to validate the gene expression level between extremely large branch angle lines (1218 and 3078, with average branch angle of 44.12° and 47.53°, respectively) and extremely small branch angle lines (2874 and 3304, with average branch angle of 26.84° and 28.33°, respectively). Gene-specific primers were listed in Table S6. As shown in Figure 8, the expression patterns of the four candidate genes detected by qRT-PCR showed significant difference between extremely large and small branch angle lines, confirming the reliability of the association mapping results. For instance, the expression levels of *TAC1* in line 1218 and line 3078 were significantly higher than that in line 2874 and line 3304 (*P* < 0.05, Figure 8A). And the expression levels of *SGR1, SGR3*, and *SGR5* in line 1218 and line 3078 were significantly lower than that in line 2874 and line 3304 (*P* < 0.05, Figures 8B–D).

## Discussion

The MLM that accounts for population structure (Q) and kinship (K), namely, the Q+K model, is a popular and powerful method used for GWASs, and it could reasonably resolve the spurious association between traits and markers caused by population structure (Yu et al., 2006; Bradbury et al., 2007). In this study, the same conclusion, i.e., the Q+K model being selected as the first-rank model, was drawn by comparing the different models (Figure S2). The Bonferroni correction is one of the typical multiple test corrections used for the threshold value of a significance test. However, it is often too conservative, such that many important loci may not pass the stringent criterion of significance test. A similar situation existed in the present study: when a GWAS was performed using the BLUP values in an MLM based on a modified Bonferroni threshold of *p* < 5.0 × 10^−5^ [−log_10_(*p*) = 4.3, 1/19,945], only one significant SNP on the A5 chromosome was discovered (Figure S1). Thus, to detect as many association signals as possible for use in further research, the significance threshold of association analysis in the MLM was dropped to a less stringent value (i.e., *p* < 1.0 × 10^−3^, −log_10_(*p*) = 3.0, Figure 5, Figure S1), which has been widely used in association mapping in rapeseed (Cai et al., 2014; Hatzig et al., 2015; Raman et al., 2015).

To prove the association results produced by the MLM and to take more advantage of the phenotypic and genotypic information obtained from an enormous amount of accessions and SNPs in this study, another model, a so-called MRMLM, was employed for a GWAS (Wang et al., 2016b). As a result, an additional 38 significance loci were identified using MRMLM, in which more than 55% of the loci overlapped part or most of the region with those obtained using MLM (Table S5), demonstrating the reliability of association analysis consequences and the practicality of combining MLM and MRMLM to improve the power and robustness of association analysis. Nevertheless, there are two prominent features in MRMLM compared to MLM. First, the MRMLM method treats marker effects as random. One advantage of this approach is that the model will shrink the effects of markers that are independent of target traits toward zero, leading to a maximum correlation between the observed and predicted phenotypic values (Goddard et al., 2009). Second, multiple test correction is not required due to the multi-locus and shrinkage nature. The MLM method is a single-locus analysis approach, in which only one marker is tested at a time. Thus, a Bonferroni correction for multiple tests is required to control the experimental error. In particular, when the number of markers is extremely large, the Bonferroni correction will be so stringent that many false-negative loci are introduced, which are significantly associated with traits in fact. Therefore, the MRMLM provides an alternative to GWASs in virtue of the power in QTL detection and the precision in locus effect estimation.

Two or more tightly related SNPs in strong LD were assigned to haplotype blocks, which were separated by recombination regions and defined the genetic variation across the genome. The block structure analysis will provide insight into the vital functional genomic regions in the course of selection and evolution (Qian et al., 2014). Therefore, genome-wide sweeping across the association panel using a high-throughput SNP chip was implemented for haplotype block structure analysis. One of the important conclusions was given based on our analysis: the large haplotype blocks were mostly distributed on the C-subgenome and were enriched around the centromere regions (Figure 3, Table 5), which is consistent with previous articles (Qian et al., 2014; Sun et al., 2016b). Here, we intend to give a plausible explanation of this phenomenon. First, the superficial reason is that the considerably stronger retention of LD leads to more long-range haplotype blocks on the C-subgenome (Qian et al., 2014). However, the ultimate contributor is the lack of genetic diversity in the C-subgenome. During Chinese *B. napus* breeding, the interspecific hybridization with *B. rapa* improves the genetic recombination and genetic diversity of the A-subgenome (Qian et al., 2006; Chen et al., 2007). However, the efforts to diversify the C-subgenome genetic component through *B. napus* × *B. oleracea* crosses were constrained due to cross-incompatibility (Bennett et al., 2008). Second, the transposon-rich regions often represent the recombination-poor (Gorelick, 2003). Recently, (Mason et al., 2016) observed the peak in transposable element density and the troughs in gene density in the centromere regions (Mason et al., 2016), which implies that lower frequency recombination events have occurred in centromere regions. Furthermore, considerably greater expansion of transposable elements was found in the C-subgenome of rapeseed (Chalhoub et al., 2014).

As depicted in previous reports, GWASs have been employed for branch angle research in rapeseed (Liu et al., 2016; Sun et al., 2016a). Hence, we compared the association consequences in this study with previous works. Unfortunately, the alignment results indicate that no identical SNP was found among them. But, encouragingly, there were nine SNPs detected by Sun et al. (2016a) that were within or proximate to loci detected in our study (Table S7). For example, two SNPs identified in the published literature on A7, Bn-A07-p15007983, and Bn-A07-p15505090, were within the locus on A7 with a peak SNP Bn-A07-p14798978 in the present paper. However, some loci detected by previous studies were still not discovered in our study, which may be affected by environmental factors, such as the location and year. In this study, the broad-sense heritability (*H*^2^) of branch angle was 76.06% (Table S2), hinting that environmental factors have a certain extent influence on the branch angle variation. Furthermore, the population size also has an important impact on the detection power of loci in GWASs, especially for rare alleles (Huang et al., 2012; Huang and Han, 2014; Li et al., 2016a). For branch angle, extensive variations are mainly caused by the cumulative effects of numerous polygenes with small effect (Sun et al., 2016a); the alleles with large effects may become rare, even extinct, in the gene pools of modern cultivars because of intensive artificial selection during domestication and modern breeding (Huang and Han, 2014). In addition, models based on a discrepant algorithm will depress the consistency of the results in GWASs. For example, approximately thirty percent of genes identified in one model could not be detected in another model in the present study.

Branch angle is regulated mainly by shoot gravitropism, which is a complex multistep process including the perception of gravity, transduction of the gravity signal into a biochemical signal, transport of the biochemical signal to a response site, and organ curvature (Sang et al., 2014). In the recent decade, many genes controlling the branch angle have been identified. *LAZY1* plays a negative role in polar auxin transport and regulates the shoot gravitropism by which the rice tiller angle is controlled (Li et al., 2007). *TAC1*, a major gene involved in branch (tiller) angle and leaf angle control in plants, has been extensively studied (Yu et al., 2007; Ku et al., 2011; Dardick et al., 2013; Zhao et al., 2014). *TAC1* and *LAZY1* are both part of the same *IGT* gene family, but the gene structures of *TAC1* and *LAZY1* differ due to the presence of an additional EAR repression motif, which has the function of transcriptional repression, in the *LAZY1* gene (Dardick et al., 2013). The difference in gene structure between *TAC1* and *LAZY1* may result in a discrepancy in molecular function; for example, there is no evidence that *TAC1* plays a role in polar auxin transport thus far, leading to *tac1* mutants with more vertical branch (tiller) angle in rice and *Arabidopsis* (Yu et al., 2007; Dardick et al., 2013). In the present study, the expression levels of *TAC1* in large branch angle lines were significantly higher (Figure 8A), suggesting that the gene functions universally to promote the horizontal growth of branches. A series of *Arabidopsis sgr* mutants have been shown to exhibit disturbed shoot gravitropism. For example, loss-of-function of *SGR5* in *Arabidopsis* and its ortholog in rice *LPA1* displays less vertical branch (tiller) angle, in which the distribution of auxin was affected through regulation of auxin biosynthesis and transport (Cui et al., 2013; Wu et al., 2013). In the present study, the expression levels of *SGR5* in small branch angle lines were significantly higher (Figure 8C), meaning that the gene is contrary to *TAC1* in the function of branch angle regulation. The polarization of PIN-mediated auxin transport leads to changes in branch angle in the *Arabidopsis* and rice (Rakusova et al., 2011; Chen et al., 2012), demonstrating the central role of auxin and auxin transport in branch growth angle control.

The genes involved in branch angle control play an important role in modulating plant architecture mostly through auxin-dependent gravitropism. In this paper, many genes for branch angle, including *TAC1, SGR1, SGR3*, and *SGR5*, were first identified in rapeseed. Although extensive studies in branch angle genes have been done, the modulation basis underlying branch angle formation and maintenance is still elusive. Digby and Firn (1995) put forward the concept of gravitropic set-point angle (GSA), defined as the growth angle with respect to gravity. Recently, Roychoudhry et al. (2013) proposed a model for GSA maintenance based on the antagonistic interaction of auxin-dependent gravitropism and the anti-gravitropic offset component (AGO), the magnitude of which is regulated by gravity sensing cells in the shoot via Aux/IAA-TIR1-ARF-dependent auxin signaling. The model provided a conceptual framework for understanding GSA variation. However, the gravitropic-AGO model may not be a case of another class of growth angles, where the organ in question is not being actively maintained relative to gravity, such as the higher order secondary branches in peach trees (Dardick et al., 2013). In particular, in rice, there is no clear evidence indicating that the already cloned genes control the tiller angle through the gravity response, except for *LAZY1* and *LPA1* (Wu et al., 2016). It is suggested that there may be some other patterns regulating branch growth angle in plants. Therefore, more thorough research is required to elucidate the molecular mechanism underlying branch angle.

## Author contributions

HL, ZL, and XW conceived and designed the study. BC, KX, and GG organized the implementation of field trials. LZ, FZ, HL, and TZ performed the phenotyping measurements. HL wrote the paper, JH, ZL, and XW modified the manuscript. All the authors have read and approved the publication of the manuscript.

### Conflict of interest statement

The authors declare that the research was conducted in the absence of any commercial or financial relationships that could be construed as a potential conflict of interest.

## Supplementary material

Figure S1 Manhattan plot of association analysis for branch angle using BLUP value in Q+K model. The horizontal blue line indicates the suggestive threshold [Bonferroni-corrected threshold −log_10_(*p*) = 4.3].The horizontal red line represents the significance threshold [−log_10_ (*p*) = 3.0].

Figure S2 Quantile–quantile plot of estimated −log_10_(*p*) from association analysis using six methods for branch angle. The black line represents the expected *p*-values with no association existed.

Table S1 Significant difference test in different branch region.

Table S2 Variance components and broad-sense heritability of branch angle.

Table S3 Haplotype block structure in rapeseed genome across 472 association population.

Table S4A Significant associated SNPs for branch angle in MLM.

Table S4B Significant associated SNPs for branch angle in MRMLM.

Table S5A Significant associated loci and candidate genes for branch angle in MLM.

Table S5B Significant associated loci and candidate genes for branch angle in MRMLM.

Table S6 Primers used for qRT-PCR.

Table S7 A comparison of the loci in the present paper and the SNPs in reference.
