# Supplementary figures and images for: Genome-Wide Association Mapping Reveals the Genetic Control Underlying Branch Angle in Rapeseed (Brassica napus L.)
Source: Front Plant Sci. 2017 Jun 19;8:1054. doi: 10.3389/fpls.2017.01054 (PMC5474488; doi:10.3389/fpls.2017.01054)

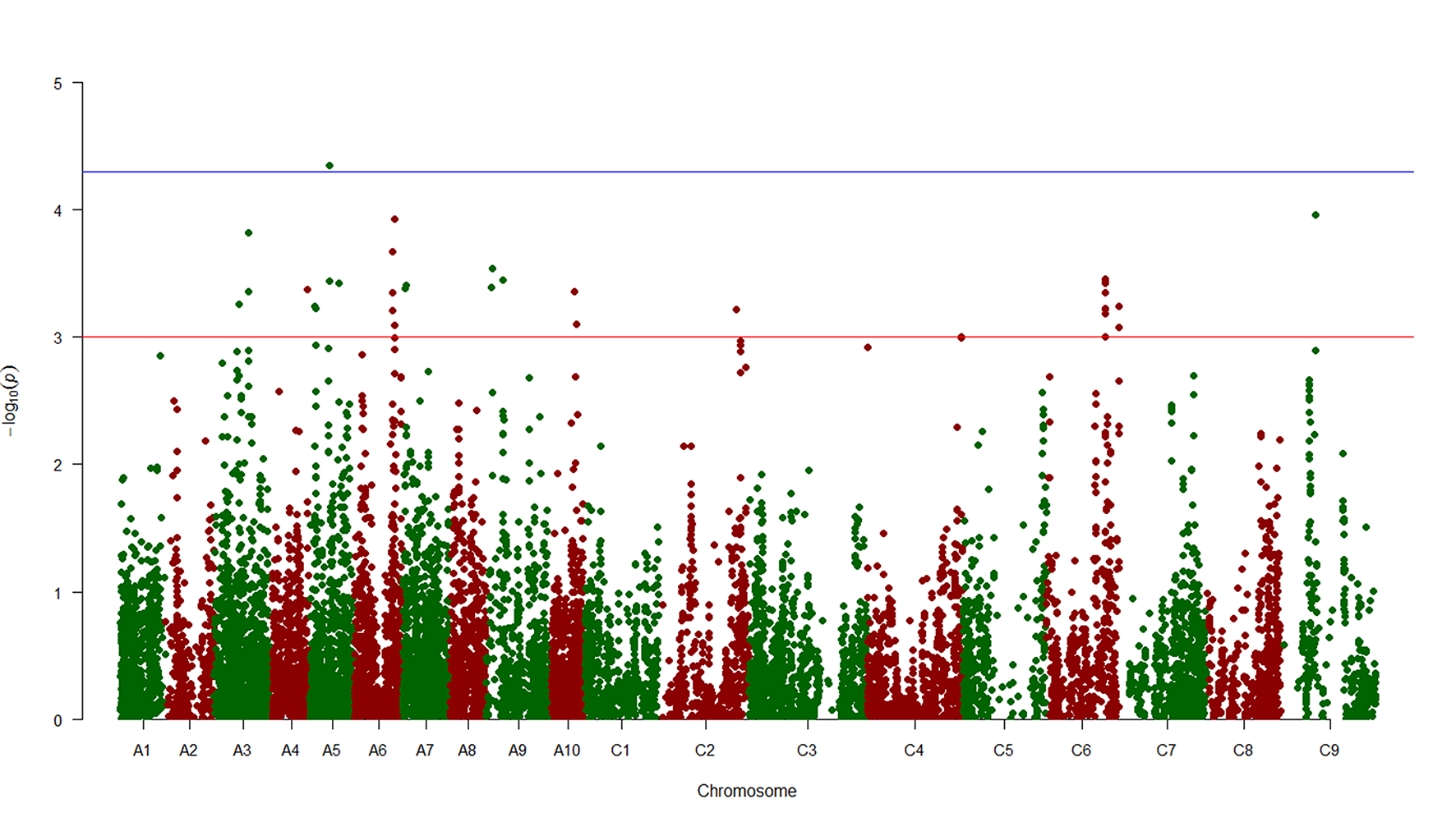

Supplement: Figure S1 — Manhattan plot of association analysis for branch angle using BLUP value in Q+K model. The horizontal blue line indicates the suggestive threshold [Bonferroni-corrected threshold −log10(p) = 4.3].The horizontal red line represents the significance threshold [−log10 (p) = 3.0]. [file Image1.TIF]

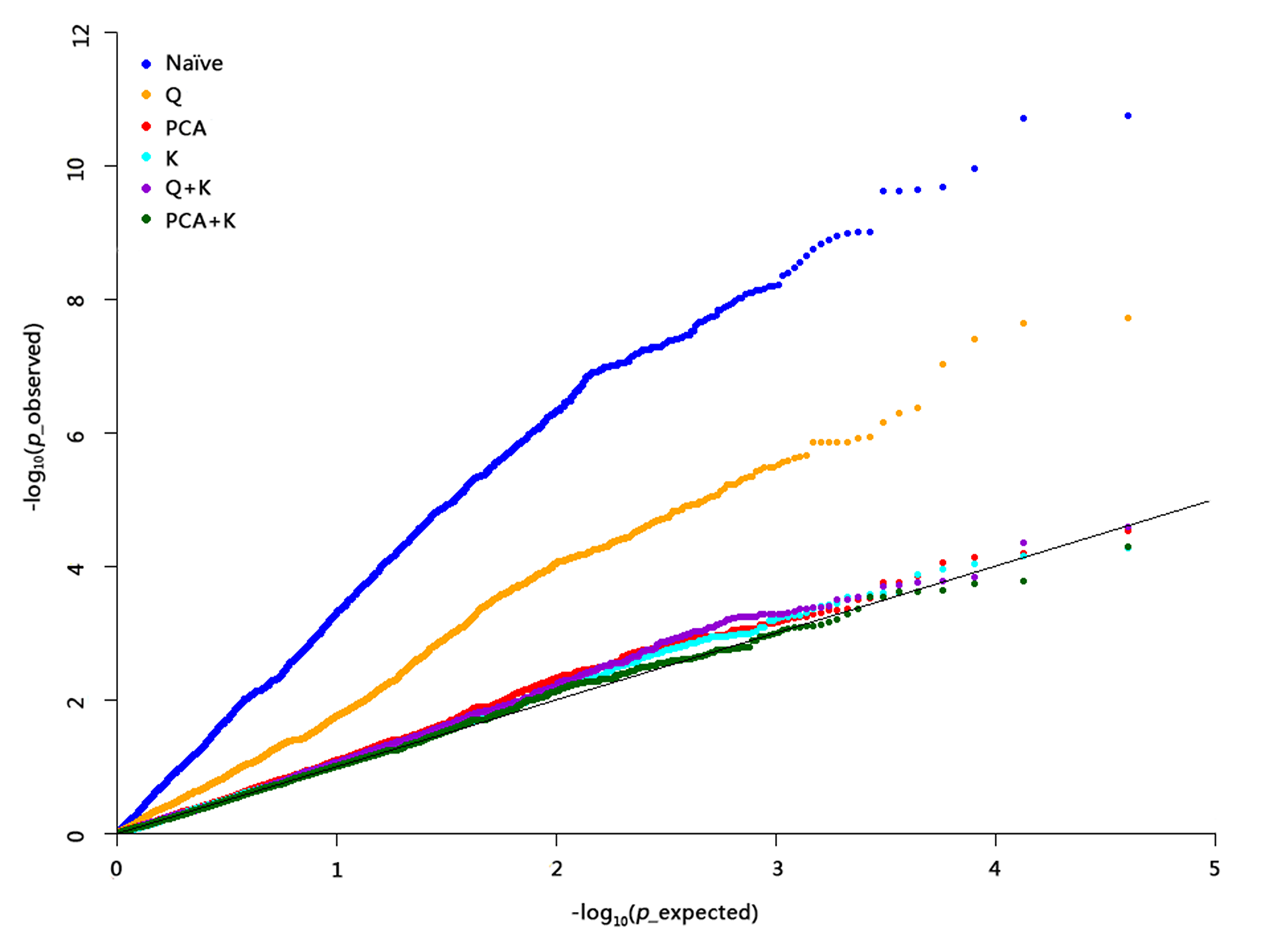

Supplement: Figure S2 — Quantile–quantile plot of estimated −log10(p) from association analysis using six methods for branch angle. The black line represents the expected p-values with no association existed. [file Image2.TIF]
